# Oral Health-Related Quality of Life in Native and Immigrant Populations in the PELFI Study in Spain

**DOI:** 10.3390/ijerph16101796

**Published:** 2019-05-21

**Authors:** Andrés A. Agudelo-Suárez, Annie M. Vivares-Builes, Natalia Muñoz-Pino, José Miguel Martínez-Martínez, Alison Reid, Elena Ronda-Pérez

**Affiliations:** 1 Faculty of Dentistry, University of Antioquia, Medellín 050010, Colombia; annievivares@gmail.com (A.M.V.-B.); munozpi88@gmail.com (N.M.-P.); 2 Public Health Research Group, University of Alicante, 03690 Alicante, Spain; elena.ronda@ua.es; 3 Research and Analysis Service, IT/EP. MC Mutual, 08029 Barcelona, Spain; jmiguelmartinezmartinez@gmail.com; 4 Department of Statistics, Technical University of Catalonia, 08034 Barcelona, Spain; 5 School of Public Health, Curtin University, Perth 6845, Australia; Alison.Reid@curtin.edu.au; 6 Centre for Biomedical Network Research on Epidemiology and Public Health (CIBERESP), 28029 Madrid, Spain; 7 Center for Research in Occupational Health (CISAL), Universitat Pompeu Fabra, 08003 Barcelona, Spain

**Keywords:** dental health surveys, emigrants and immigrants, oral health, quality of life

## Abstract

Quality of life and its relationship to oral health is an important consideration in the determinants of health of vulnerable groups. The aim of this study is to assess oral health-related quality of life (OHRQoL) and its related factors in native and immigrant population families from the Platform of Longitudinal Studies on Immigrant Families (PELFI) study in Spain. A cross-sectional study was conducted in a sample of 401 adults aged 18 years and older from Spain, Ecuador, Colombia, and Morocco. The OHIP-14 instrument was applied, and three summary variables were used (prevalence, extent, and severity). Sociodemographic and self-perceived health variables were included. Bivariate analyzes were carried out to summarize the variables of the OHIP-14 according to sociodemographic and health variables, and bivariate analyzes of the OHIP-14 dimensions was conducted by country of origin. Multivariate linear models were used to investigate predictors for the dimensions of the OHIP-14. Multivariate logistic models were used to estimate the association of OHRQoL with immigration status using crude and adjusted odds ratios with 95% confidence intervals (OR-95% CI). It was found that 14.8% of men and 23.8% of women reported negative impacts in terms of OHRQoL (statistically significant differences: *p* < 0.05). There were statistically significant differences according to the country of origin in the prevalence and severity outcomes of the OHIP-14 in women (*p* < 0.05), and severe outcomes were observed in Moroccan women. In women, statistically significant differences (*p* < 0.05) in OHRQoL were observed according to age and marital status. There were some differences between OHIP-14 summary outcomes according to the health variables. Some sociodemographic and health variables were predictors for the OHIP-14 and their dimensions with differences by sex. Multivariate analysis showed a statistically significant association between OHRQoL and immigration status for Moroccan women. Differences in OHRQoL were found according to sociodemographic and health variables. Further research could clarify the predictors of OHRQoL through epidemiological surveillance and longitudinal studies.

## 1. Introduction

Quality of life and its relationship to health is a topic that has generated interest on the scientific agenda for several decades [1]. The operative definition of quality of life is related to human experiences and the satisfaction people feel with their situations of physical, emotional, and spiritual health and, in their family, friendship and social environments [2]. Oral health is a dimension of quality of life. Oral health-related quality of life (OHRQoL) evaluates the impact of the state of oral health and different oral pathologies on quality of life compared to elements such as dysfunction, discomfort and disability and their functional and psychological consequences [3]. One of the most commonly used instruments to evaluate OHRQoL from the perception of the individual is the Oral Health Impact Profile (OHIP), which was developed in the 1990s in a 49-question version (OHIP-49) and later in a shorter version with 14 questions, the OHIP-14 [4].

Different studies have shown the relationship between the social, economic, and cultural determinants that can give way to situations of inequality among different social groups in terms of quality of life [5,6]. A systematic review found that the social conditions most clearly associated with a negative perception of OHRQoL were being a woman with low income and a low level of education and belonging to an ethnic minority or immigrant group, among other demographic factors [7]. The literature also relates quality of life and oral health to systemic diseases [8] and risk factors such as obesity and malnutrition taking into account body mass index [9] and the relationship between OHRQoL and general and mental health [10].

Spain has experienced a population transformation due to the migration of people mainly from low-income countries. Their motivations for migration are economic and labor-related which provides Spain with a unique migratory profile, and the demographic profile of the country has undergone significant changes. Although there was a significant increase in the foreign-born population in the years 2000–2008, the economic crisis beginning in 2009 resulted in important effects on the working conditions and health conditions of this population [11]. Many of these immigrants returned to their countries of origin. Those in better situations, especially those with employment and with nationality or other work permits, have remained with their families in Spain [12].

While there are studies that evaluate the impact of quality of life on oral health in immigrant groups in other contexts [13,14,15,16], studies that focused on the working immigrant population are scarce. In Spain, in particular, there are only epidemiological studies on oral health in children. These studies compare caries between immigrant children and native children [17,18], and there is a study that evaluates the impact of oral health on the quality of life in a group of immigrant and Spanish pregnant women [19]. In general, the studies indicate that the impact of oral health on quality of life could be greater among immigrant groups, taking into account other factors such as the time since migration, socioeconomic aspects, and determinants related to access to health services [7]. In Spain, several policies instigated since 2012 have restricted access to health care for undocumented immigrants. The health system is influenced by insurance status which is granted by employers who provide work permits [20]. Access to oral health services is paid privately and therefore inequalities in the use of dental care services can arise [21]. Evaluating all of the aspects related to occupational health of the immigrant population is important, including oral health.

In 2014, the Platform of Longitudinal Studies on Immigrant Families (PELFI) was established as a multi-centric cohort of immigrant families in Spain. The goal of this project was to be able to include cohorts from different Spanish cities and to obtain information to study the history and impact of migration on health [22]. This project included questions that allowed for the evaluation of the self-perceived oral health, the use of oral health and dental services among the immigrant working population, and the impact of oral health on quality of life. The objective of this current study is to determine the OHRQoL of the immigrant and native population over age 18 in the PELFI study and to identify related sociodemographic factors.

## 2. Materials and Methods

### 2.1. Study, Sampling Design, and Data Collection

This is a cross-sectional analysis from a prospective cohort study. Data were drawn from the PELFI project. The project comprises a prospective multi-site cohort study drawn from a convenience sample in order to access the hard-to-reach population of immigrant families of those countries of origin with greatest representation in Spain. The recruitment began in 2015, and additional details of the methodology are described elsewhere [22]. A statistical power analysis was performed with the program GRANMO 7.12 (Institut Municipal d’Investigació Mèdica, Barcelona, Spain). To summarize, 250 families were recruited through the use of key informants, with an enrollment rate of 82%; within each family, every adult (i.e., ≥18 years) and every adolescent (i.e., 12–17 years) were interviewed. They were interviewed in their homes, or at associations or public places in the neighborhood, by professional, trained interviewers. A pilot study was carried out with 18 families, in order to review the quality of the survey and ensure the understanding of the questions. Specifically, for this analysis, data from the first and second waves were used and 401 adults (females: n = 146; 61.3%) from the sub-cohorts of Alicante (n = 192) and Barcelona (n = 209) were selected. They were born in Spain (n = 101), Ecuador (n = 126), Colombia (n = 122), and Morocco (n = 52). Data were collected through a structured questionnaire which can be consulted at the following webpage: https://web.ua.es/es/gi-saludpublica/trabajo-inmigracion-y-salud-en-una-cohorte-de-poblacion-inmigrante-en-espana.html.

### 2.2. Variables

The outcome of interest was OHRQoL. For this purpose, the OHIP-14 instrument was used [4]. The OHIP-14 is a shortened version of a scale and includes 14 questions to assess seven dimensions of the impact of oral conditions on people’s quality of life: functional limitation, physical pain, psychological discomfort, physical disability, psychological disability, social disability, and handicap. Responses are recorded on a five-point ordinal scale coded (0 = never, 1 = hardly ever, 2 = occasionally, 3 = fairly often, 4 = very often). Table 1 shows the seven dimensions of the OHIP-14 instrument with their respective questions. The questionnaire is available in Spanish and it has been validated for use in epidemiological studies of the working population in Spain [23].

Data provided for the questions of the OHIP-14 were used to calculate three summary indicators as used previously in the literature [24]: (1) Prevalence: the percentage of respondents reporting one or more impacts “fairly often” or “very often”. This variable identifies those whose oral health impacts are chronic rather than transitory; (2) Extent: the number of items reported “fairly often” or “very often”; (3) Severity: the sum of the response codes for the 14 items. This takes into account impacts experienced at all levels of frequency. Given the response codes, severity scores range from 0 to 56, with higher values indicating more frequent impacts. Additionally, for each dimension, the response codes were summed with a range from 0 to 8. Higher impacts in the three summary variables are related to lower overall OHRQoL.

The following sociodemographic variables were considered: sex, age (18–34, 35–44, 45–54, and ≥55), education level (≤primary, secondary, and ≥university), marital status (single, married/cohabitating, and other), social class (no manual/manual) [25], country of origin (Spain, Ecuador, Colombia, and Morocco).

To evaluate health status, certain general and oral health indicators were used separately: (1) self-rated general health (current-prior): categorized as good (good/very good) or poor (fair/poor/very poor); (2) body mass index (BMI): defined as a person’s weight in kilograms divided by the square of his/her height in meters (kg/m^2^), and with this information and according to the parameters of the WHO [26], the following characteristics were determined: (a) underweight: BMI ≤ 18.50; (b) normal weight: BMI between 18.50 and 24.99; (c) overweight obesity: BMI ≥ 25.00. For this case, the self-reported BMI was used, which means that this measure was based on the responses of the surveyed people about their weight and height; (3) mental health: this variable were assessed by the 12-item General Health Questionnaire and is a well-validated measure of common mental disorders to detect current, non-psychotic disorders, patterns of adjustment associated with distress. For each item, a score of 0 (for responses 1 and 2, less symptomatic) or 1 (for answers 3 and 4, more symptomatic) was assigned and the 12 resulting scores were added together. Responses scoring ≥3 were classified as poor mental health) [27]; (4) self-perceived dental caries (no/unknown/yes); (5) self-perceived gingival bleeding (no/unknown/yes); (6) use of oral health services (<1 year and ≥1 year/never).

### 2.3. Statistical Analysis

Analyses were carried out separately for men and women. The complexity of the interaction of social determinants of health cannot be understood entirely without taking into account gender, a factor of social stratification. SPSS software version 22.0-IBM^®^ (SPSS Inc., Chicago, IL, USA) was used to carry out the analyses.

First, absolute and relative frequencies were calculated for each of the sociodemographic and health variables. Second, a bivariate analysis was conducted for each of the summary measures of the OHIP-14 questionnaire: prevalence (%), extent (average and standard deviation), and severity (mean, standard deviation, median, and interquartile range), according to the sociodemographic and health variables. Tests of statistical significance were carried out to observe differences among variables (Mann–Whitney U test for dichotomous variables, Kruskal–Wallis test for polychotomous variables in the case of non-parametric data, and the Chi-square test for the distribution of frequencies for categorical variables). The seven dimensions that make up the OHIP-14 were explored (mean, standard deviation, median, and interquartile range) by country of origin, and significance tests were calculated related to the nature of the variables (non-parametric data).

A linear multivariate regression analysis was carried out to evaluate the simultaneous and reciprocal effect of the sociodemographic/health variables on each of the OHIP-14 dimensions, at the extent and severity scores for identifying possible predictors. Belonging was determined by evaluating the compliance with the assumptions of linearity, non-collinearity and normality, constant variance, and correlation of residuals. All of the analyses used a level of statistical significance of <0.05.

Finally, a multivariate analysis was carried out using logistic regression to observe the association between migration status (exposure variable) and the summary variable “prevalence” (the probability of reporting one or more impacts “fairly often” or “very often”). Crude and adjusted odds ratios and 95% confidence intervals were calculated.

### 2.4. Ethics Approval and Consent to Participate

Ethical approval of the PELFI project was obtained from the ethical committees of the University of Alicante (Act UA-2014-06-26) and the Vall d’Hebron Research Institute. Confidentiality was guaranteed throughout the research process, and all respondents gave informed consent to participate in accordance with Spanish regulations.

## 3. Results

The sociodemographic characteristics of the sample and the three summary indicators of the OHIP-14 are presented in Table 2. Fifteen percent of men and 24 percent of women reported negative impacts in the OHRQoL (statistically significant differences between both sexes, *p* < 0.05). The mean global scores of severity were 6.2 (±7.8) for men and 7.7 (±9.1) for women. The summary indicators analyzed (prevalence, extent, and severity) were higher for all women compared with those for all men.

In men (Table 2), the prevalence and severity indicators were higher in men from a non-manual labor social class and among Moroccans. These indicators differed when age and marital status were considered. For women, statistically significant differences were found for the prevalence indicators according to sociodemographic factors, except for the variable age. When the total OHIP-14 score was analyzed (severity), there were significant differences except for social class. Both indicators (prevalence and severity) were greater among people with primary or a lesser education level, with “other” civil status (widowed, separated), among people with a manual social class and in Moroccan women.

Table 3 presents the summary indicators of the OHIP-14 by general and oral health variables. For both men and women, the three indicators (prevalence, extent, and severity) were highest for those people who reported that their current and prior health was poor, who reported overweight or obesity, and those with dental caries or gingival bleeding. However, when use of oral health services was considered, in the case of men, the indicators (prevalence, extent, and severity) were greater in those whose last visit was less than 1 year ago, while for women the indicators were greater for those who used oral health services ≥1 year ago or never used the services. There were statistically significant differences in the case of women who reported poor health indicators, caries, and gingival bleeding.

Table 4 presents the different dimensions of the OHIP-14 instrument, by country of origin. Moroccan women reported greater mean and median scores, compared with those in the other countries, especially for the dimensions of physical pain and psychological discomfort. In women, there were statistically significant differences when the different dimensions were compared between countries, with the exception of the dimension functional limitation. In men, while the scores were greater in Moroccan men, there were no statistically significant differences when the dimensions were compared between countries.

The results of the multivariate linear regression (Table 5) show that, in men, the variable self-perceived gingival bleeding was a predictor for the majority of the dimensions and of the extent and severity scores of OHIP-14. The exception was for the functional limitation dimension, for which the predictive variable was self-rated general health (prior). In the psychological disability dimension, the variable self-rated general health (prior) was also a predictor. Education level was also a predictor for the social disability dimension. In women, the predictors for the OHRQoL were self-rated general health (prior), self-rated general health (current), and self-perceived gingival bleeding. Country of origin was a variable predictor for the physical pain dimension. Marital status was a predictor for extent and physical disability. The variable mental health was a predictor of the psychological disability dimension, and finally the social disability dimension was predicted by the variable use of oral health services but in a negative way. The independent variables described explained between 3.2 percent and 25 percent of the scores obtained for the dimensions, for the extent outcome and for the global total for men and women.

Table 6 presents the results of the multivariate analysis related to the prevalence indicator and according to migration status. The probability of reporting one or more impacts “fairly often” or “very often” of oral health on quality of life is five times greater in Moroccan women (OR: 5.08; 95% CI: 1.93–13.34), which remained after adjusting for health variables (Model 3). Statistical significance was lost when it was adjusted by sociodemographic variables (Model 2) and it remained at *p* < 0.10 when adjusted by oral health variables (Model 4).

## 4. Discussion

The principal findings of this study show how the general scores from the OHIP-14 instrument which are related to the OHRQoL were low among the surveyed population, which shows that oral health has a low impact on quality of life (that is to say, good quality of life related to oral health). However, there were differences by sociodemographic factors, general and oral health. Furthermore, there were differences in the perception of quality of life between men and women for some variables. Moroccan women reported a greater impact of oral health on quality of life (in the global scores, for different dimensions and for the prevalence indicator). Women had greater scores in the physical pain and psychological discomfort dimensions with statistically significant differences by country of origin. The general health and oral health variables were predictive of up to one quarter of the dimension and extent scores and of the global scores for the OHIP-14 instrument (severity).

This study found the summary indicators for oral quality of life differed by sex. In general terms, women reported greater prevalence, extent and severity, and this situation was maintained when sociodemographic and health indicators were compared for the analysis of the OHIP-14 by dimensions. The literature shows how social determinants and the principal axes of inequality vary by sex [28]; and in this case, there are situations that are unfavorable for women with the lowest levels of education and foreign women (especially those from Morocco). Similar results were observed in the study carried out by the WHO Collaborating Center for Disability, Culture and Oral Health, National Center for Transcultural Oral Health, where women reported a greater impact of oral health on quality of life, especially related to its social and psychological aspects [29]. Possible explanations for these findings include different self-care practices related to the roles of men and women [30] and a greater need for healthcare among women for biological and social reasons [31]. Similarly, considering the characteristics of the study population, it is important to analyze gender inequalities related with educational opportunities, marital strain, caregiving responsibilities, positions in the labor market, and work-related exposures and subsequent health effects [32].

Migration status was an axis of inequality in the quality of life indicators related to oral health. In the case of the immigrant population from Ecuador and Colombia, the quality of life related to oral health was better for some indicators, in contrast to that of the Moroccan population. A systematic review found that immigrants, especially those with less time since immigration, reported more negative impacts related to the OHRQoL, for example, related to country of birth, socioeconomic status, general health status, and access to oral health services [13]. Explanations for these findings relate to the effect of the area of residence of both sub-cohorts in Alicante and Barcelona, given that the study was carried out in areas of high social deprivation. Other studies have shown how area of residence is an important axis of inequality in terms of social conditions, health conditions, quality of life, and access to health care for the immigrant population [33,34].

The literature provides broad coverage of the “healthy immigrant effect”, in which the immigrant population shows better health than the local population, caused by the selective migration of people in good health. Oral health is no exception, although the indicators for the OHRQoL differ by immigrant group, and the situation is a bit better for Ecuadorians and Colombians than for Moroccans. This loss of the healthy immigrant effect in terms of oral health has been shown in a longitudinal study carried out in Canada [35]. That study shows how the risk of oral health problems increased over the time of follow-up. In addition, the social determinants play a role that regulate the oral health and the quality of life in different social groups and is influenced by the cultural context of individuals [7].

Oral health responds not only to biological factors of the individual. There are also factors related to the social and economic context that influence the epidemiological profile and quality of life, especially for people who are considered vulnerable. It is important to mention the relationship between quality of life, oral health, and general health [14], given that the OHRQoL indicators tend to be higher in those people who report worse general health and oral health indicators. In the same way, there are different dimensions that have more of an effect on quality of life, such as those related to physical pain and psychological discomfort that affect people’s daily routines [36].

This is supported by the multivariate linear regression analysis, which found that health variables (including mental health) and oral health (especially gingival bleeding) were predictive variables that influence to a greater extent the impact on oral health and quality of life (or worse OHRQoL scores). Even though results are not easily comparable due to the characteristics of the PELFI sample, a study carried out in North Carolina (U.S.) among agricultural immigrants shows a relationship between the number of functional oral health problems and OHRQoL indicators measured via the OHIP-14 and health and quality of life indicators measured via the SF-36 [10].

One of the strengths of this study is that this is the first time to follow the immigrant population in Spain that is based on the family unit as the fundamental element for analyzing health conditions. This study also obtained response rates favorable to the study objectives. The instruments used to collect information were carefully designed to ensure internal consistency and were pilot-tested [22]. The OHIP-14 instrument was validated in Spain and has been used previously with the working population [23].

A limitation of the study is that the recruitment of families was carried out using a convenience sample, which could result in selection bias, since many people were invited to participate through associations and through snowball sampling. It is possible that people were excluded who had less social support or worse economic conditions. This could result in underestimation of the study results. However, it is important to note that probabilistic sampling is also incapable of ensuring a representative sample in studies related to immigration and health, as shown by prior research [37,38]. The cross-sectional nature of this analysis limited the ability to make causal inferences, although the results were consistent with other similar studies. Longitudinal prospective studies could be useful to identify changes in the OHRQoL, including other variables related to the migratory process. The study included immigrant families from three foreign countries: Ecuador, Colombia, and Morocco. They were chosen on the basis of having a greater presence in Spain and considering budget constraints, which reduced the possibilities of including families on from other countries. The Spanish language requirement within the inclusion criteria for this study means that the sample may focus on a subset of immigrants who are already more acculturated than those who do not speak the language.

While recognizing these limitations, this study permits an approach to the study of the OHRQoL in the immigrant and Spanish population according to sociodemographic and health factors. Further research should focus on identifying oral health conditions and their determinants using clinical indicators. In the same way, the use of qualitative studies should not be discarded. These studies allow for the exploration of perceptions related to oral health and quality of life, as has taken place in other geographic contexts [39].

## 5. Conclusions

The general scores related to the OHRQoL are low among the immigrant and Spanish population included in the study, which shows a relatively good quality of life related to oral health. However, there are differences according to sociodemographic and health variables. The identification of factors related to OHRQoL should permit the development of health promotion and prevention strategies based on the social reality of the population in addition to incorporation of oral illnesses in epidemiological surveillance systems, in order to effectively monitor social inequalities in oral health.

## Figures and Tables

**Table 1:** Dimensions and questions for the OHIP-14 used in the PELFI cohort, Spain (n = 401).

Dimension	Questions
Functional limitation	Have you had trouble pronouncing any words because of problems with your teeth or mouth?
	Have you felt that your sense of taste has worsened because of problems with your teeth or mouth?
Physical pain	Have you had painful aching in your mouth?
	Have you found it uncomfortable to eat any foods because of problems with your teeth or mouth?
Psychological discomfort	Have you been self-conscious because of your teeth or mouth?
	Have you felt tense because of problems with your teeth or mouth?
Physical disability	Has your diet been unsatisfactory because of problems with your teeth of mouth?
	Have you had to interrupt meals because of problems with your teeth or mouth?
Psychological disability	Have you found it difficult to relax or sleep because of problems with your teeth or mouth?
	Have you been a bit embarrassed because of problems with your teeth or mouth?
Social disability	Have you been a bit irritable with other people because of problems with your teeth or mouth?
	Have you had difficulty doing your usual jobs because of problems with your teeth or mouth?
Handicap	Have you felt that life in general was less satisfying because of problems with your teeth or mouth?
	Have you been totally unable to function because of problems with your teeth or mouth?

**Table 2:** Summary outcomes of the OHIP-14 as a proxy of oral health-related quality of life by sociodemographic variables in the PELFI cohort, Spain (n = 401).

Variables	Sample		Oral Health-Related Quality of Life (Summary Outcomes of OHIP-14)			
	n	%	Prevalence (%)	Extent (X ± SD) ^a^	Severity	
					(X ± SD) ^a^	Me (IQR) ^b^
**Males**						
Age (years)						
18–34	26	16.8	23.1	0.8 (1.8)	6.3 (9.3)	2.0 (9.0)
35–44	45	29.0	15.6	0.4 (1.1)	6.4 (7.4)	5.0 (9.0)
45–54	75	48.4	10.7	0.4 (1.3)	5.6 (7.6)	3.0 (8.0)
≥55	9	5.8	22.1	0.3 (0.7)	9.8 (6.9)	9.0 (12.0)
Education level						
≤Primary	36	23.2	11.1	0.5 (1.7)	6.7 (8.1)	4.5 (8.0)
Secondary	87	56.1	16.1	0.4 (1.3)	5.9 (8.0)	2.0 (9.0)
≥University	32	20.6	15.6	0.4 (1.1)	6.1 (7.0)	4.5 (10.0)
Marital status						
Single	21	13.5	14.3	0.6 (1.7)	6.5 (9.3)	2.5 (9.0)
Married/cohabited	131	84.5	15.3	0.4 (1.3)	6.2 (7.6)	4.0 (9.0)
Other	3	1.9	---	---	2.7 (2.3)	4.0 (--)
Social class						
Non-manual	16	13.8	31.3	0.6 (1.1)	6.8 (5.7)	4.0 (10.0)
Manual	100	86.2	13.0	0.3 (1.1)	5.6 (6.6)	4.0 (10.0)
Country of origin						
Spain	44	28.4	15.9	0.3 (0.7)	6.3 (6.5)	5.0 (8.0)
Ecuador	44	28.4	9.1	0.4 (1.3)	6.1 (8.3)	4.0 (9.0)
Colombia	46	29.7	13.0	0.2 (0.7)	4.5 (5.7)	2.0 (8.0)
Morocco	21	13.5	28.6	1.4 (2.6)	9.6 (11.8)	4.0 (15.0)
**Total**	**155**	**100.0**	14.8 *	0.4 (1.3) *	**6.2 (7.8)**	**4.0 (9.0)**
**Females**						
Age (years)						
18–34	44	17.9	15.9	0.4 (1.1)	5.1 (7.4) *	2.0 (6.0) *
35–44	103	41.9	21.4	0.6 (1.5)	7.0 (8.0) *	4.0 (11.0) *
45–54	89	36.2	28.1	1.0 (2.2)	9.9 (10.4) *	5.5 (16.0) *
≥55	10	4.1	30.0	0.7 (1.6)	7.6 (9.2) *	3.5 (17.0) *
Education level						
≤Primary	58	23.6	34.5 *	1.3 (2.4) *	10.7 (11.2)	8.0 (20.0)
Secondary	136	55.3	21.3 *	0.5 (1.4) *	6.5 (8.2)	4.0 (10.0)
≥University	52	21.1	15.4 *	0.5 (1.6) *	7.7 (8.1)	4.5 (10.0)
Marital status						
Single	50	20.3	26.0 **	0.7 (1.6) **	8.4 (9.9) *	4.0 (13.0) *
Married/cohabited	157	63.8	17.2 **	0.5 (1.6) **	6.6 (8.2) *	4.0 (10.0) *
Other	39	15.9	43.6 **	1.5 (2.3) **	11.7 (10.4) *	10.0 (19.0) *
Social class						
Non-manual	19	9.8	0.0 *	---	6.2 (5.7)	4.0 (10.0)
Manual	175	90.2	19.4 *	0.6 (1.6) *	7.0 (8.7)	4.0 (10.0)
Country of origin						
Spain	57	23.2	19.3 ***	0.7 (1.7) ***	8.1 (7.8) **	6.0 (11.0) **
Ecuador	82	33.3	20.7 ***	0.6 (1.7) ***	7.3 (9.1) **	3.5 (11.0) **
Colombia	76	30.9	15.8 ***	0.5 (1.4) ***	5.6 (8.1) **	3.0 (7.0) **
Morocco	31	12.6	54.8 ***	1.6 (2.4) ***	13.5 (11.2) **	15.5 (21.0) **
**Total**	**246**	**100.0**	23.2 *	0.7 (1.7) *	**7.7 (9.1)**	**4.0 (12.0)**

^a^ (X ± SD): mean ± standard deviation. ^b^ Me: median; IQR: interquartile range. Mann–Whitney/Kruskal–Wallis test for non-parametric data and Chi-square test for categorical variables: * *p* < 0.05, ** *p* < 0.01, *** *p* < 0.001.

**Table 3:** Summary outcomes of the OHIP-14 as a proxy of oral health-related quality of life by health variables in the PELFI cohort, Spain (n = 401).

Variables	Sample		Oral Health-Related Quality of Life (Summary Outcomes of OHIP-14)			
	n	%	Prevalence (%)	Extent (X ± SD) ^a^	Severity (OHIP-14 Score)	
					(X ± SD) ^a^	Me (IQR) ^b^
**Males**						
Self-rated general health (current)						
Good	96	61.9	14.6	0.3 (1.0)	5.2 (6.3) *	3.0 (8.0)
Poor	59	38.1	15.3	0.6 (1.8)	7.8 (9.6) *	5.0 (11.0)
Self-rated general health (prior)						
Good	107	70.4	10.3 **	0.3 (1.4) *	5.2 (6.6) *	3.0 (7.0)
Poor	45	29.6	24.4 **	0.8 (1.9) *	8.5 (9.9) *	4.0 (13.0)
Body Mass Index						
Normal	69	44.5	10.1	0.4 (1.3)	5.9 (8.0)	3.0 (9.0)
Underweight	0	0.0	---	---	---	---
Overweight Obesity	86	55.5	18.6	0.5 (1.4)	6.4 (7.6)	4.0 (10.0)
Mental health (GHQ-12)						
Good	77	49.7	14.3	0.4 (1.1)	5.0 (6.7)	2.0 (8.0)
Poor	78	50.3	15.4	0.5 (1.5)	7.3 (8.6)	4.0 (12.0)
Self-perceived dental caries						
No/Unknown	111	71.6	11.5	0.3 (1.1)	5.1 (6.5)	2.0 (8.0)
Yes	44	28.4	21.6	0.7 (1.7)	8.3 (9.6)	5.0 (12.0)
Self-perceived gingival bleeding						
No/Unknown	111	71.6	10.8 *	0.2 (0.6) *	4.3 (5.4) **	2.0 (7.0) **
Yes	44	28.4	25.0 *	1.1 (2.2) *	11.0 (10.5) **	8.0 (15.0) **
Use of oral health services						
<1 year	68	44.2	17.6	0.5 (1.3)	6.3 (7.6)	4.0 (10.0)
≥1 year/never	86	55.8	12.8	0.4 (1.3)	6.0 (8.0)	3.0 (9.0)
**Females**						
Self-rated general health						
Good	125	51.0	16.0 **	0.4 (1.3) **	5.3 (7.4) **	2.0 (8.0) **
Poor	120	49.0	36.8 **	1.0 (2.1) **	10.2 (10.0) **	7.0 (15.0) **
Self-rated general health (prior)						
Good	177	72.5	15.3 ***	0.4 (1.1) ***	5.9 (7.5) ***	3.0 (9.0) ***
Poor	67	27.5	44.8 ***	1.6 (2.6) ***	13.0 (10.9) ***	12.0 (19.0) ***
Body mass index						
Normal	103	41.9	18.4	0.6 (1.7)	6.5 (9.0) *	3.0 (8.0) *
Underweight	2	0.8	0.0	----	2.0 (2.8) *	2.0 (---) *
Overweight-Obesity	141	57.3	27.0	0.8 (1.8)	8.8 (9.1) *	5.0 (15.0) *
Mental health (GHQ-12)						
Good	107	43.5	16.8 *	0.5 (1.5)	6.0 (8.1) **	3.0 (9.0) **
Poor	139	56.5	28.1 *	0.8 (1.9)	9.0 (9.6) **	5.5 (15.0) **
Self-perceived dental caries						
No/Unknown	185	75.2	14.6 ***	0.4 (1.0) ***	5.6 (7.0) ***	3.0 (9.0) ***
Yes	61	24.8	42.7 ***	1.5 (2.6) ***	12.7 (11.3) ***	10.0 (18.0) ***
Self-perceived gingival bleeding						
No/Unknown	185	75.2	17.3 ***	0.5 (1.3) ***	5.8 (7.2) ***	3.0 (9.0) ***
Yes	61	24.8	41.0 ***	1.4 (2.6) ***	13.7 (11.3) ***	11.0 (17.0) ***
Use of oral health services						
<1 year	122	49.6	20.5	0.6 (1.6)	7.3 (8.7)	4.0 (11.0)
≥1 year/never	124	50.4	25.8	0.8 (1.9)	8.2 (9.5)	4.0 (13.0)

Missing values for the following variables: self-rated general health (prior), n = 5; and use of oral health services, n = 1. ^a^ (X ± SD): mean ± standard deviation. ^b^ Me: median; IQR: interquartile range. Mann–Whitney/Kruskal–Wallis test for non-parametric data and Chi-square test for categorical variables: * *p* < 0.05, ** *p* < 0.01, *** *p* < 0.001.

**Table 4:** Dimensions of the OHIP-14 according to country of origin in the PELFI cohort, Spain (n = 401).

OHIP-14 Dimensions	Spain		Ecuador		Colombia		Morocco		*p*-Value ^c^
	X (±SD) ^a^	Me (IQR) ^b^	X (±SD) ^a^	Me (IQR) ^b^	X (±SD) ^a^	Me (IQR) ^b^	X (±SD) ^a^	Me (IQR) ^b^	
**Males**									
Functional limitation	0.4 (0.8)	0.0 (0.0)	0.4 (1.2)	0.0 (0.0)	0.4 (0.9)	0.0 (0.0)	0.7 (1.8)	0.0 (1.0)	0.475
Physical pain	1.8 (1.7)	2.0 (4.0)	1.5 (1.8)	1.0 (2.0)	1.3 (1.6)	0.0 (2.0)	2.0 (2.3)	1.5 (4.0)	0.377
Psychological discomfort	1.6 (1.8)	1.0 (3.0)	1.4 (2.1)	0.0 (2.0)	1.3 (1.9)	0.0 (2.0)	1.8 (2.7)	0.0 (3.0) *	0.604
Physical disability	0.9 (1.3)	0.0 (2.0)	1.0 (1.6)	0.0 (2.0)	0.7 (1.3)	0.0 (1.0)	2.3 (2.6)	1.0 (4.0)	0.477
Psychological disability	1.0 (1.4)	0.0 (2.0)	0.9 (1.5)	0.0 (2.0)	0.5 (1.0)	0.0 (1.0)	1.4 (1.9)	0.0 (2.0)	0.266
Social disability	0.3 (0.7)	0.0 (0.0)	0.3 (0.9)	0.0 (0.0)	0.2 (0.6)	0.0 (0.0)	0.6 (1.3)	0.0 (0.0)	0.530
Handicap	0.4 (0.9)	0.0 (0.0)	0.7 (1.6)	0.0 (0.0)	0.2 (0.7)	0.0 (0.0)	1.0 (1.7)	0.0 (2.0)	0.090
**Females**									
Functional limitation	0.4 (0.8)	0.0 (1.0)	0.7 (1.2)	0.0 (0.0)	0.4 (1.0)	0.0 (0.0)	0.9 (1.6)	0.0 (5.0)	0.275
Physical pain	2.1 (1.9)	2.0 (4.0)	1.6 (1.8)	1.0 (3.0)	1.3 (1.6)	1.0 (2.0)	2.9 (2.5)	3.0 (3.0)	0.028
Psychological discomfort	2.1 (2.2)	2.0 (4.0)	1.6 (2.1)	0.0 (3.0)	1.5 (2.0)	0.0 (2.0)	3.3 (2.5)	3.0 (3.0) *	0.001
Physical disability	1.6 (2.1)	0.0 (3.0)	1.5 (1.9)	0.0 (4.0)	0.8 (1.7)	0.0 (0.0)	2.5 (2.2)	2.0 (4.0)	<0.001
Psychological disability	1.1 (1.2)	1.0 (2.0)	1.1 (1.6)	0.0 (2.0)	1.0 (1.8)	0.0 (2.0)	1.8 (1.8)	2.0 (3.0)	0.014
Social disability	0.4 (0.8)	0.0 (0.0)	0.4 (1.0)	0.0 (0.0)	0.3 (1.0)	0.0 (0.0)	1.2 (1.7)	0.0 (2.0)	0.008
Handicap	0.5 (1.0)	0.0 (1.0)	0.4 (1.4)	0.0 (0.0)	0.3 (0.9)	0.0 (0.0)	1.3 (1.7)	0.0 (3.0)	<0.001

^a^ (X ± SD): mean ± standard deviation. ^b^ Me: median; IQR: interquartile range. ^c^ Mann–Whitney/Kruskal–Wallis test for non-parametric data comparing countries and OHIP-14 dimensions in the sample. (* statistically significant differences between men and women, *p* < 0.05).

**Table 5:** Lineal regression models for the scores of dimensions of the OHIP-14 in the PELFI cohort, Spain (n = 401).

Dimension	Males			Females		
	Model Variable	Regression Coefficient	Determination Coefficient (%)	Model Variable	Regression Coefficient	Determination Coefficient (%)
Extent	Self-perceived gingival bleeding	0.38 ***	14.0	Marital status	0.216 **	23.0
				Self-rated general health (prior)	0.285 ***	
				Self-rated general health (current)	0.186 **	
				Self-perceived gingival bleeding	0.173 **	
OHIP-14 (Severity)	Self-perceived gingival bleeding	5.6 ***	13.0	Self-rated general health (prior)	5.870 ***	25.0
				Self-rated general health (current)	3.961 ***	
				Self-perceived gingival bleeding	6.289 ***	
Functional limitation	Self-rated general health (prior)	0.532 *	5.0	Self-rated general health (prior)	0.438 **	9.5
				Self-perceived gingival bleeding	0.687 ***	
Physical pain	Self-perceived gingival bleeding	1.320 ***	9.6	Country of origin	0.533 *	21.0
				Self-rated general health (prior)	1.059 ***	
				Self-rated general health (current)	0.649 **	
				Self-perceived gingival bleeding	1.412 ***	
Psychological discomfort	Self-perceived gingival bleeding	1.411 **	9.3	Self-rated general health (prior)	1.629 ***	21.3
				Self-rated general health (current)	0.929 **	
				Self-perceived gingival bleeding	1.230 ***	
Physical disability	Self-perceived gingival bleeding	0.647 *	3.2	Marital status	0.481 *	19.7
				Self-rated general health (prior)	1.110 ***	
				Self-rated general health (current)	0.909 **	
				Self-perceived gingival bleeding	1.044 **	
Psychological disability	Self-rated general health (prior)	0.607 *	9.1	Self-rated general health (prior)	0.761 **	19.0
				Self-rated general health (current)	0.786 ***	
	Self-perceived gingival bleeding	0.649 *		Mental health (GHQ-12)	0.454 *	
				Self-perceived gingival bleeding	0.750 **	
Social disability	Educative level	0.207 *	7.5	Self-rated general health (prior)	0.505 **	12.4
				Self-rated general health (current)	0.296 *	
	Self-perceived gingival bleeding	0.321 *		Self-perceived gingival bleeding	0.469 *	
				Use of oral health services	−0.274 *	
Handicap	Self-perceived gingival bleeding	1.184 *	19.7	Self-rated general health (prior)	0.383 *	10.0
				Self-rated general health (current)	0.348 *	
				Self-perceived gingival bleeding	0.698 ***	

* *p* < 0.05, ** *p* < 0.01, *** *p* < 0.001.

**Table 6:** Association between the prevalence outcome of the OHIP-14 and country of origin by means of multivariate analysis in the PELFI cohort, Spain (n = 401).

Multivariate Logistic Analysis for the Prevalence Outcome of the OHIP-14	Model 1	Model 2	Model 3	Model 4
	OR (95% CI)	OR (95% CI)	OR (95% CI)	OR (95% CI)
***Males***				
Spain	1.00	1.00	1.00	1.00
Ecuador	0.53 (0.14–1.95)	0.80 (0.10–6.58)	0.52 (0.12–2.15)	0.31 (0.10–1.34)
Colombia	0.79 (0.24–2.58)	1.65 (0.22–12.17)	0.75 (0.18–3.05)	0.60 (0.16–2.19)
Morocco	2.11 (0.61–7.34)	1.72 (0.14–21.33)	2.10 (0.54–8.22)	1.67 (0.42–6.75)
***Females***				
Spain	1.00	1.00	1.00	1.00
Ecuador	1.09 (0.47–2.56)	1.40 (0.45–4.35)	1.29 (0.49–3.39)	0.78 (0.31–1.99)
Colombia	0.78 (0.32–1.93)	1.07 (0.29–3.96)	0.94 (0.35–2.57)	0.79 (0.31–2.02)
Morocco	5.08 (1.93–13.34) ***	1.31 (0.18–9.75)	5.25 (1.78–15.51) ***	3.11 (1.00–9.70) *

Model 1: unadjusted (crude OR). Model 2: adjusted OR by age, education, marital status, and social class. Model 3: adjusted OR by health variables: self-rated general health (current-prior), body mass index, and mental health (GHQ-12). Model 4: adjusted OR by all oral health variables: self-perceived dental caries, self-perceived gingival bleeding, and use of oral health services. * *p* < 0.10, ** *p* < 0.05, *** *p* < 0.01.
